# 

**Published:** 1899-02

**Authors:** 


### BOOKS RECEIVED.

The Treatment of Wounds. Its Principles and Practice, General and Special. By L. S. Pilcher, A. M., M. D., Surgeon to the Methodist-Episcopal Hospital in New York, etc., etc. One volume, 8vo, 466 pages. Profusely illustrated. Muslin, $3.00 net. New York: William Wood & Co.

The Medical News Pocket Formulary for 1899. Containing 1,600 prescriptions representing the latest and most approved methods of administering remedial agents. By E. Quin Thornton, M. D., Demonstrator of Therapeutics, Pharmacy and Materia Medica in the Jefferson Medical College, Philadelphia. In one wallet-shaped volume, strongly bound in leather, with pocket and pencil. Price, $1.50 net. Philadelphia and New York: Lea Brothers & Co., Publishers.

Saunders's Pocket Medical Formulary, with an Appendix. By William M. Powell, M. D., Author of Essentials of Diseases of Children; Associate Editor Annual Universal Medical Sciences, etc. Fifth edition, thoroughly revised. Price, $1.75 net. Philadelphia: W. B. Saunders. 1899.

A Text-Book of Mechano-Therapy (Massage and Medical Gymnastics.) Especially prepared for the use of medical students and trained nurses. By Axel V. Grafstrom, B. Sc., M. D., late Lieutenant in the Royal Swedish Army; late House Physician City Hospital, Blackwell's Island, New York. With eleven pen-and-ink sketches by the author. Price, $1.00. Philadelphia: W. B. Saunders. 1899.

The Practice of Obstetrics: By American Authors. Edited by Charles Jewett, M. D., Professor of Obstetrics in Long Island College Hospital, Brooklyn, N. Y. In one handsome 8vo volume of 736 pages, with 441 engravings in colors and black, and twenty-two full-page colored plates. Cloth, $5.00 net; leather, $6.00 net. Philadelphia and New York: Lea Brothers & Co., Publishers. 1899.

Annual and Analytical Cyclopedia of Practical Medicine. By Charles E. de M. Sajous, M. D., and 100 associate editors. Assisted by corresponding editors, collaborators and correspondents. Illustrated with chromo-lithographs, engravings and maps. Volume II. Bromide of Ethyl to Diphtheria. Philadelphia, New York and Chicago: F. A. Davis Co. 1899.

Report of the Commissioner of Education for the Year 1896-97. Volume II. Containing Parts II. and III. Washington: Government Printing Office. 1898.

Twentieth Century Practice. An International Encyclopedia of Modern Medical Science. By leading authorities of Europe and America. Edited by Thomas L. Stedman, M. D., New York City. In twenty volumes. Volume XVII. Infectious Diseases and Malignant Neoplasms. New York: William Wood & Co. 1898.

Formulaire des Médicaments Nouveaux pour 1899, par H. Bocquillon-Limousin, pharmacien de 1re classe, lauréat de l'École de pharmacie de Paris. Introduction par le Dr. Huchard, médecin des hôpitaux. 1 vol. in-18 de 324 pages, cartonné. 3 fr. Paris: Librairiè J. B. Baillière et Fils. 1899.

The American Year-Book of Medicine and Surgery. Being a yearly digest of scientific progress and authoritative opinion in all branches of medicine and surgery, drawn from journals, monographs and text-books of the leading American and foreign authors and investigators. Collected and arranged with critical editorial comments by eminent American specialists and teachers. Under the general editorial charge of George M. Gould, M. D. Illustrated. Philadelphia: W. B. Saunders. 1899.

